# The Early Impact of the COVID-19 Lockdown on Stress and Addictive Behaviors in an Alcohol-Consuming Student Population in France

**DOI:** 10.3389/fpsyt.2021.628631

**Published:** 2021-02-09

**Authors:** Valentin Flaudias, Oulmann Zerhouni, Bruno Pereira, Cheryl J. Cherpitel, Jordane Boudesseul, Ingrid de Chazeron, Lucia Romo, Sébastien Guillaume, Ludovic Samalin, Julien Cabe, Laurent Bègue, Laurent Gerbaud, Benjamin Rolland, Pierre-Michel Llorca, Mickael Naassila, Georges Brousse

**Affiliations:** ^1^CHU Clermont-Ferrand, Pôle Psychiatrie B, Clermont-Ferrand, France; ^2^Université Clermont Ferrand, EA NPsy-Sydo, BP 10448, Clermont-Ferrand, France; ^3^Laboratoire Parisien de Psychologie Sociale, Département de Psychologie, University Paris Nanterre, Ad Hoc Lab, Nanterre, France; ^4^Alcohol Research Group, Emeryville, CA, United States; ^5^Facultad de Psicología, Instituto de Investigación Científica, Universidad de Lima, Lima, Peru; ^6^EA4430 CLIPSYD, UFR SPSE, Paris Nanterre University, Nanterre, France; ^7^CMME, GHU Paris Psychiatrie et Neurosciences, U de Paris, Paris, France; ^8^Department of Emergency Psychiatry and Post-Acute Care, CHRU Montpellier/INSERM U1061, University of Montpellier, Montpellier, France; ^9^LIPC2S, Université Grenoble Alpes, Grenoble, France; ^10^Service de Santé Publique, CHU de Clermont-Ferrand, Clermont-Ferrand, France; ^11^Université Clermont Auvergne, CNRS-UMR 6602, Institut Pascal, Axe TGI, Groupe PEPRADE, Clermont-Ferrand, France; ^12^Service Universitaire d'Addictologie de Lyon (SUAL), Pôle MOPHA, CRNL, Inserm U1028, CNRS UMR5292, Université Lyon 1, Centre Hospitalier Le Vinatier, Bron, France; ^13^Université de Picardie Jules Verne, Unité INSERM UMR 1247, Groupe de Recherche sur l'Alcool & les Pharmacodépendances, Centre Universitaire de Recherche en Santé, Amiens, France

**Keywords:** COVID-19, Coronavirus, stressors, LockDown, addiction, alcohol, public health

## Abstract

**Background:** This study evaluated factors linked with perceived stress related to the COVID-19 pandemic and lockdown and addictive behaviors prior to and during lockdown in a sample of students who indicated engaging in alcohol consumption behaviors before lockdown.

**Methods:** Cross-sectional study. French students from four universities participated in this study, and 2,760 students reported alcohol use. During the first week of lockdown, students reported their perceived levels of stress regarding COVID-19. Substance use and addictive behaviors were reported before and during lockdown, and media exposure, demographical, living conditions, and environmental stressors were reported during lockdown.

**Results:** Women reported greater levels of stress (95% CI: 1.18 to 1.93, *p* < 0.001). Highly-stressed students also report less social support (95% CI: −1.04 to −0.39, *p* < 0.001) and were more likely to worry about the lockdown (95% CI: 0.27 to −0.65, *p* < 0.001). Alcohol-related problemswere more prevalent among the most stressed students (95% CI: 0.02 to 0.09, *p* = 0.004) as well as eating problems (95% CI: 0.04 to 0.36, *p* = 0.016) and problematic internet use (95% CI, 0.06 to 0.14, *p* < 0.001). Students reporting the highest levels of stress also indicated more compulsive eating during the previous seven days (95% CI, 0.21 to 1.19, *p* = 0.005).

**Conclusions:** The level of stress was strongly related to four categories of variables: (i) intrinsic characteristics, (ii) addictive behaviors before lockdown, (iii) lockdown-specific conditions, and (iv) addictive behaviors during the lockdown. Several variables linked to COVID-19 were not directly linked with perceived stress, while perceived stress was found to correlate with daily life organization-related uncertainty and anticipated consequences of lockdown. Importantly, social support seems to be a protective factor on high level of stress.

## Introduction

As of September 13, 2020, at least 917,417 confirmed deaths and more than 28,637,952 cases of infections by Coronavirus disease 2019 (COVID-19) have been reported worldwide (1). Persistence of the disease is observed globally, with a resurgence of cases in Europe (11% more new cases over the last 7 days at the time of writing this article). Technical guidance^1^ and public policies have varied across countries. However, about a third of the human population have been advised or constrained to stay home except for essential activities, and as a result nearly three billion people have endured lockdown (1). While pandemics are primarily a physical health concern, they also have a massive impact on social and mental health. During a lockdown characterized by uncertainty regarding the future, being unable to have a normal personal and interpersonal life creates an unstable and potentially anxiety-producing and threatening environment (2, 3). Public health concern regarding the potential detrimental effects of long-term lockdowns on mental health therefore have recently surged in interest (4).

In particular, issues linked to alcohol consumption are of primary importance; previous scientific claims having indicated the risk of a significant public health crisis in the future due to increased alcohol consumption during the lockdown (5–7). Perceived stress is indeed known to be an important factor in the development and maintenance of an alcohol use disorder, particularly among young adults (8). A recent French study showed that the COVID-19 lockdown was associated with a substantial proportion of participants reporting increased intake of high-caloric or salty food as well as online activity and consumption of tobacco, alcohol, and cannabis (9). Furthermore, these individuals shared several additional features, including increased stress. Consistent with this, recent data on a French sample from an European study (10) showed that psychological distress occurred in a third of respondents during lockdown. However, vulnerability to the epidemic (e.g., susceptibility to contracting COVID-19) did not appear to be a major determinant of psychological distress during the lockdown. Because a rapid daily environment degradation can have a negative impact on mental health (11, 12), this sudden increase in environmental pressure causing major uncertainties and adverse emotional experiences is likely to promote potentially harmful coping strategies and foster risky behavior.

College students are particularly vulnerable to stress-related disorders (12) or addictive disorders (13). Currently, a large body of literature has shown that students are at high risk for alcohol abuse and alcohol use disorder (14, 15). In addition, college students are at a particularly precarious time of their life (16–18), with limited financial resources and therefore likely to be living in stressful and perhaps highly dense housing conditions during the lockdown. Moreover, university students have had to adapt to an unprecedented shift in remote teaching and exams, which has also likely contributed to increasing their perceived stress level. As a result, they are at an increased risk of developing addictive behaviors, particularly problematic alcohol consumption (18). Students who use alcohol have been shown to be at greater risk of developing an addiction when exposed to daily stressful situations (19). However, to our knowledge, no studies have examined the addiction-related behaviors of students who use alcohol during an intensely stressful event.

Here, we evaluate the perceived stress related to the COVID-19 pandemic and lockdown in a sample of students who indicated being alcohol consumers before lockdown.

We are interested in the effect of lockdown-induced stress on students' drinking behavior. In view of the effects of stress on self-regulatory behavior, high stress should be associated with an increase in alcohol consumption among students, but not necessarily with the emergence of addictions to new substances (20, 21). Thus, this population is particularly at risk of developing self-regulation difficulties in stressful situations. Recent theories of self-regulation do not make it possible to identify the extent to which these self-regulation difficulties could influence other addictive behaviors in this population. For this, we assessed factors associated with perceived stress and addictive behaviors prior to and during lockdown.

It was hypothesized that during the first week and the 15 subsequent days of lockdown after the survey, addictive behaviors would be associated with the level of perceived stress related to the COVID-19 pandemic and the lockdown, but also to addictive behaviors as assessed prior to lockdown.

We conducted a survey in a population of students who indicated they engaged in alcohol consumption prior to lockdown, and assessed (i) characteristics of participants, conditions of the lockdown and the resulting change in lifestyle and social support; (ii) characteristics of students' addictive behaviors before lockdown; (iii) perceived stress related to fear induced by COVID-19, the conditions of the lockdown and exposure to media; (iv) levels of anxiety and depression during this period; and (v) addictive behaviors during lockdown. Specifically, we explored alcohol, tobacco and cannabis consumption in addition to gaming, internet use and problematic eating behaviors (compulsion or restriction) during the first week of lockdown and the intention the following 15 days after the survey. Furthermore, we explored whether student profiles would appear as a function of their level of perceived stress, with variables of interest contributing the most to different levels of perceived stress, thus allowing us to identify potential risk factors.

## Methods

### Participants and Procedure

The present study was an ancillary project drawn from a larger database; this database was previously examined (22) to show the impact of stress factors induced by COVID-19 on problematic eating behaviors for all students in the database. An online questionnaire was sent to students of four French universities (University of Clermont Auvergne, University of Picardie Jules Verne, University of Paris Nanterre and University of Grenoble-Alpes) and distributed over a single 2-day period, from 26 to 27 March 2020 (The beginning of the lockdown was declared on 17 March, 2020). The STROBE guidelines were used to ensure the reporting of this cross-sectional study (23).

Students were contacted via the university digital work environment of the University Clermont Auvergne (37,367 students), the University of Picardie (30,288 students), and Paris Nanterre (500 psychology students). The survey was also shared on the Facebook page, “University of Grenoble Alpes” (4,626 views). The number of students potentially targeted by this survey was 72,781. All participants responded anonymously. Since there is no strict exclusion criterion in the literature on alcohol consumption and since we tried to have the broadest sample possible, our inclusion criterion was all participants who drink alcohol occasionally or regularly (24). Participants were asked the question “Do you drink alcohol at all? Participants who answered “yes” were then given the AUDIT and the questions on alcohol consumption. Only students who reported drinking were included in the analyses. This study was approved by the Ethics Committee of the University of Clermont Auvergne.

### Measures

The online questionnaire gathered the following data: Sociodemographic characteristics (characteristics included age, gender, whether the student had a scholarship (for financial need) and level of education), level of social support, perceived stress, level of anxiety and depression, lockdown and COVID-19-specific information, addictive behaviors before lockdown, and addictive behaviors during lockdown. Table 1 describes the instruments used to obtain these data.

**Table 1:** Description of assessments used in this study.

**Dimensions**	**Scale**	**Description**	**Range score**
Social support	Social provisions scale scores, SPS10 (25)	The SPS10 assesses five forms of social provisions with 10 items: attachment, guidance, social integration, reliable alliance, and reassurance of worth. Each item is rated on a four-point Likert scale	A continuous scale score is computed from the responses to the 10 questions. Higher scores can be interpreted as indicating higher levels of social support
Perceived stress	A French version of the visual analog scale of the Perceived Stress scores, PSS10 (26–28)	The PSS10 evaluates the degree to which an individual perceives life as unpredictable, uncontrollable and overloading. The PSS10 also assesses the degree to which external demands seem to exceed the individual's perceived ability to cope	A score on the scale below 21 indicates that the person knows how to manage stress (less stress group), while a score between 21 and 26 indicates that the person knows most of the time how to manage stress (mild stress group). A score above 27 indicates that life is a perpetually threatening environment for the person (high stress group). We used the same categorization adapted to the version of the scale used in this study (*inferior to 32.5 for low, between 32.5 and 65 include for mild stress group and superior to 65 for high stress group*) (26)
Level of anxiety and depression	Hospital Anxiety and Depression scale, HADS (29)	The HADS is a 14-item measure of state-anxiety and depression	Each item is scored from 0 to 3, with higher scores indicating greater anxiety or depression
Lockdown and COVID-19-specific information (see Appendix A for more details)	- Two scales were developed: (i) A specific, 13-item scale of stressors associated with COVID-19 and the lockdown (ii) A media exposure to COVID-19 and health information scale (5 items). - Data on conditions of lockdown was also assessed	(i) This scale assesses specific lockdown concerns (11 items) and concerns about being infected by COVID-19 for oneself or loved ones (2 items). (ii) This scale assesses specific media exposure to COVID 19 - Condition of lockdown included the number of children under 12 and the number of adults with whom the respondent is confined and the type of their housing (personal housing with no roommates, apartment-sharing, university dormitories, at their parents' house). Having a loved one infected, hospitalized or deceased because of COVID-19 was also accessed (This score is calculated from 0 to 3 by summing each category)	(i) This scale is rated from 0 to 6 per item, with 0 being the lowest stress level and 6 the highest. An average score is calculated by the mean of the rate of each item. The total score ranges from 0 to 6. (ii) This scale is rated from 0 to 4 per item, with 0 being the lowest stress level and 4 the highest. The total score ranges from 0 to 4. An average score is calculated
Addictive behaviors before lockdown	- Fagerström test (30) for tobacco, - Alcohol Use Disorder test (AUDIT) scores for alcohol (31), - Cannabis Abuse Screening Test (CAST) (32) for cannabis, - SCOFF (33) for food compulsion and restriction - body dissatisfaction and impulse regulation subscales of the Eating Disorder Inventory, 2nd edition (EDI2) (34), - Internet Gaming Disorder Scale (IGDT10) (35) and the Compulsive Internet Use Scale (CIUS) (36) for internet use disorders	Validated scales	Higher scores indicating greater problematic addictive behavior
Addictive behaviors during lockdown (see Appendix B for more details)	A self-developed questionnaire about Addictive behaviors during lockdown	Behaviors were determined using a developed questionnaire about the quantity of substance used on a daily or weekly basis (alcohol, tobacco, or cannabis). Data on time spent playing and/or being on the internet as well as eating habits were collected for the past 7 days and on participants'intentions for the next 15 days	Higher scores indicating greater problematic addictive behavior.

### Statistical Analyses and Measures

First, descriptive analyses were performed, and only students who reported drinking were included in subsequent analyses. Descriptive analyses were performed according to the level of perceived stress assessed with the PSS10, which was categorized into three groups: low (score inferior or equal to 32.5), medium (score between 32.5 and 65 included), and high stress (score superior to 65). To assess the impact on student stress levels, demographic and other characteristics described above were compared for medium and high stress groups with the low stress group, using a univariate mixed-effects multinomial logistic regression with university as random effect to consider variability between and within each university. Then, to evaluate a model in which all the variables can significantly modulate the level of perceived stress, multivariable analysis was carried out, and covariates were selected according to univariate results and clinical relevance. For multiple comparisons, variables were included in the multivariable regression (i.e., the multilevel mixed-effect multinomial logistic model) when they were significant in univariate for a type I error at 0.005. Close attention was paid to examining multicollinearity and interactions between covariates: (1) studying the relationships between the covariables, (2) estimating the variance inflation factor, and (3) measuring the impact of adding or removing variables in the multivariable model. For the multivariable analysis, we set the level of significance at 0.05, applying a Sidak's type I error correction due to multiple comparisons (low stress vs. medium and low stress vs. high). The results were expressed as coefficients and 95% confidence intervals.

Finally, multidimensional analyses as a factorial mixed data analysis (FMDA) were performed (i) to illustrate student profiles according to the level of perceived stress and (ii) to highlight potential factors associated with perceived stress. These statistical methods were useful for analyzing assets as elements of qualitative and quantitative variables in order to uncover the underlying relationships and structures of the variables measured (latent constructs) and to aggregate subjects into clusters such that each cluster represents a topic.

Analyses were performed with Stata 15.0 (StataCorp, College Station, US) for random-effects models and software R (package ade4) for factorial analyses.

## Results

In total, 5,738 students (women = 74.2%, mean age = 21.2, SD = 5.17) from four French universities participated in this study (see Table 2). The response rate of the survey was 7.9%. Two thousand seven hundred sixty students reported alcohol use (48% of the total sample) and were included in subsequent analyses (women = 70,1%, men = 21.3, SD = 4.71).

**Table 2:** Intrinsic characteristics of the study participants.

	**Intrinsic characteristics**
	**Mean (SD) or** ***n*** **and %**
*N*	5,671
Age	21.2 (4.50)
**Gender**
Women	1,431 (25.4%)
Men	4,210 (74.6%)
**Scholarship**
Yes	2,766 (48.8%)
No	2,905 (51.2%)
**Education levels (only** ***N*** **were reported) compared with 1st year**
1st year (L1)	1,862 (32.8%)
2nd year (L2)	963 (17%)
3rd year (L3)	979 (17.3%)
4rth year (M1)	586 (10.3%)
5th year (M2)	478 (8.4%)
Doctorate/PhD	177 (3.1%)
Advanced Technical or Marketing Degree (BTS/DUT)	353 (6.2%)
IUT (3-year course–University Institute of Technology)	273 (4.8%)
SPS10	3.38 (0.482)
HADSA	8.97 (4.35)
HADSD	5.6 (3.56)
**PSS**
Low stress	1,174 (20.7%)
Mild stress	2,843 (50.1%)
High stress	1,655 (29.2%)

### Relationship of Demographic, Lockdown, and COVID-19-Specific Information, and Addictive Behaviors Before and During Lockdown With the Level of Perceived Stress

The characteristics of the population are reported in Table 3, and only variables with *p*-values below 0.005 are displayed. Five hundred and ninety-eight (22%) students had a low level of perceived stress, 1,405 (51%) had a mild level of perceived stress, while 757 (27%) had a high level of perceived stress.

**Table 3:** Participants characteristics by perceived level of stress.

				**Perceived stress scale category**							
				**Low vs. Mild**				**Low vs. High**			
	**Low mean (SD) or n and %**	**Mild Mean (SD) or n and %**	**High M (SD) or n and %**	**Coef**.	***P*****-value**		**95% CI**	**Coef**.	***P*****-value**		**95% CI**
*N*	598	1,405	757								
**Intrinsic characteristics**						
Age	21.6 (5.04)	21.3 (4.54)	21.2 (4.74)	−0.14	0.161	−0.033	0.005	−0.019	0.174	−0.037	0.006
Gender				0.951	<0.001	0.752	1.150	1.598	<0.001	1.347	1.347
Women	295 (11%)	1003 (37%)	622 (22%)								
Men	298 (11%)	393 (14%)	128 (5%)								
Scholarship				0.121	0.221	−0.072	0.315	0.320	0.004	0.103	0.536
Yes	252 (9%)	630 (23%)	378 (14%)								
No	344 (12%)	772 (28%)	378 (14%)								
**Education levels (only** ***N*** **were reported) compared with 1st year**						
1st year (L1)	155	416	239								
2nd year (L2)	93	225	120	−0.065	0.680	−0.374	0.244	−0.140	0.425	−0.482	0.203
3rd year (L3)	111	251	142	−0.129	0.391	−0.424	0.165	−0.145	0.383	−0.469	0.180
4rth year (M1)	60	171	87	0.114	0.525	−0.238	0.466	−0.007	0.971	−0.398	0.383
5th year (M2)	64	122	69	−0.304	0.097	−0.663	0.054	−0.320	0.117	−0.719	0.079
Doctorate/PhD	21	55	21	−0.033	0.905	−0.572	0.506	−0.441	0.177	−1.08	0.199
Advanced technical or marketing degree (BTS/DUT)	47	92	51	−0.323	0.116	−0.725	0.079	−0.359	0.118	−0.808	0.091
IUT (3–year course–University Institute of Technology)	47	73	28	−0.531	0.012	−0.945	−0.115	−0.934	<0.001	−1.447	−0.421
SPS10	3.57 (0.399)	3.44 (0.425)	3.28 (0.521)	−0.790	<0.001	−1.042	−0.538	−1.493	<0.001	−1.763	−1.224
HADSA	5.17 (2.74)	8.42 (3.45)	12.6 (2.95)	0.349	<0.001	0.308	0.389	0.646	<0.001	0.597	0.694
HADSD	3.36 (2.45)	5.15 (2.95)	7.96 (3.84)	0.237	<0.001	0.194	0.279	0.502	<0.001	0.454	0.550
**Addictive behaviors before lockdown**						
Fagerstrom	0.378 (0.780)	0.463 (0.841)	0.553 (0.886)	0.118	0.056	−0.003	0.240	0.239	<0.001	0.107	0.370
AUDIT	6.04 (3.80)	6.61 (4.66)	7.51 (5.57)	0.032	0.006	0.009	0.054	0.068	<0.001	0.044	0.092
SCOFF	0.883 (0.875)	1.31 (1.04)	1.80 (1.17)	0.464	<0.001	0.357	0.572	0.860	<0.001	0.742	0.978
IGTD10	2.33 (2.87)	2.72 (3.30)	3.50 (3.98)	0.038	0.017	0.007	0.068	0.098	<0.001	0.065	0.130
CIUS	6.84 (3.81)	8.59 (3.94)	9.86 (4.30)	0.113	<0.001	0.087	0.138	0.187	<0.001	0.159	0.216
CAST				0.527	0.002	0.186	0.868	0.644	0.001	0.278	1.010
Yes	46 (2%)	176 (6%)	105 (4%)								
No	550 (20%)	1226 (44 %)	651 (24%)								
**Lockdown specific scales**
Number of children present during lockdown with the participant	0.134 (0.417)	0.141 (0.485)	0.150 (0.489)	0.027	0.803	−0.183	0.237	0.067	0.567	−0.163	0.300
Number of adults present during lockdown with the participant	1.94 (1.15)	1.92 (1.06)	1.91 (1.12)	−0.012	0.787	−0.100	0.075	−0.028	0.573	−0.127	0.070
**Type of housing during lockdown (only** ***N*** **were reported) compared with personal housing**						
Personal housing (no roommates)	292	689	377								
Apartment–sharing	75	187	91	0.054	0.727	−0.248	0.355	−0.063	0.716	−0.406	0.279
University dormitories	62	97	58	−0.381	0.032	−0.729	−0.033	−0.293	0.141	−0.683	0.097
Parents' house	169	432	231	0.029	0.803	−0.200	0.259	0.006	0.962	−0.249	0.261
Media exposure	1.98 (0.570)	2.10 (0.593)	2.18 (0.654)	0.347	<0.001	0.174	0.520	0.553	<0.001	0.364	0.741
Having a loved one infected, hospitalized or deceased because of COVID−19	0.451 (0.710)	0.510 (0.705)	0.585 (0.749)		0.176	−0.044	0.240		0.003	0.082	0.389
Stressors Lockdown	2.63 (1.107)	3.33 (0.904)	3.79 (0.901)	0.692	<0.001	0.588	0.797	1.269	<0.001	1.136	1.402
Stressors COVID−19	3.25 (1.47)	3.77 (1.34)	3.99 (1.35)	0.247	<0.001	0.180	0.315	0.339	<0.001	0.260	0.417
**Addictive behaviors during lockdown**						
Drinking frequency last week	2.68 (1.33)	2.65 (1.32)	2.68 (1.32)	−0.014	0.706	−0.087	0.059	0.001	0.994	−0.081	0.082
Drinking quantity last week	1.60 (1.14)	1.82 (1.86)	2.03 (1.90)	0.091	0.025	0.012	0.170	0.110	0.011	0.026	0.194
Drinking intention next 15 weeks (Yes/No)				−0.021	0.828	−0.214	0.171	−0.033	0.763	−0.249	0.183
Yes	322 (12%)	768 (28%)	416 (15%)								
No	274 (10%)	634 (23%)	340 (12%)								
Drinking frequency intention next 15 days	1.44 (0.610)	1.49 (0.634)	1.54 (0.683)	0.133	0.213	−0.076	0.343	0.230	0.050	−0.001	0.460
Standard drinks per occasion intention	2.70 (2.39)	2.71 (3.09)	2.72 (2.36)	0.004	0.840	−0.032	0.039	0.004	0.825	−0.035	−0.035
Binge drinking frequency before confinement	1.30 (0.842)	1.27 (0.927)	1.31 (0.920)	−0.014	0.801	−0.120	0.092	0.039	0.520	−0.079	0.157
Binge drinking occurrence last week				0.055	0.870	−0.604	0.715	−0.434	0.209	−1.110	0.243
Yes	13 (less 1%)	30 (1%)	26 (1%)								
No	583 (22%)	1372 (50%)	730 (26%)								
Binge drinking frequency last week	0.0688 (0.442)	0.129 (1.68)	0.134 (0.662)	0.173	0.160	−0.068	0.414	0.176	0.157	−0.068	0.419
Virtual binge drinking (if binged last week)				−0.524	0.555	−2.262	1.214	−0.010	0.992	−1.864	1.846
Yes	2 (3%)	7 (10%)	4 (6%)								
No	11 (16%)	23 (33%)	22 (32%)								
Binge drinking intention next 15 days				−0.091	0.677	−0.518	0.336	−0.409	0.075	−0.859	0.0420
Yes	31 (1%)	80 (3%)	58 (2%)								
No	565 (21%)	1322 (48%)	698 (25%)								
Binge drinking frequency next 15 days	1.48 (1.19)	2.63 (6.96)	2.85 (3.46)	0.232	0.149	−0.083	0.548	0.239	0.138	−0.0770	0.556
Online gaming last week	29.2 (36.1)	28.8 (35.6)	33.6 (38.1)	−0.001	0.875	−0.003	0.002	0.003	0.029	0.001	0.006
Online gaming next 15 days	20.5 (29.2)	24.7 (30.9)	29.6 (34.8)	0.004	0.006	0.001	0.008	0.009	<0.001	0.006	0.012
Food compulsion last week	1.27 (0.629)	1.59 (0.843)	1.92 (1.000)	0.633	<0.001	0.478	0.788	1.007	<0.001	0.845	1.168
Food compulsion next 15 days	1.04 (0.189)	1.13 (0.415)	1.27 (0.589)	1.065	<0.001	0.646	1.485	1.607	<0.001	1.184	2.029
Food restriction last week	1.52 (0.934)	1.87 (1.09)	2.15 (1.20)	0.342	<0.001	0.239	0.444	0.556	<0.001	0.446	0.665
Food restriction next 15 days	1.59 (1.01)	1.93 (1.14)	2.28 (1.25)	0.292	<0.001	0.197	0.428	0.529	<0.001	0.387	0.631

Women comprised 49.8% (*N* = 295) of the low stress group compared with 71.85% (*N* = 1,003) in the mild stress group (95% CI, 0.75 to 1.15, *p* < 0.001) and 82.9% (*N* = 622) in the high stress group (95% CI, 1.35 to 1.85, *p* < 0.001).

#### The Mild Stress Group vs. the Low Stress Group

Compared to the low stressed students, mildly stressed students included a higher proportion of women, had a higher Hospital Anxiety and Depression scale–HADS (37) score for both anxiety (95% CI, 0.31 to 0.39, *p* < 0.001) and depression (95% CI, 0.19 to 0.28, *p* < 0.001) and indicated less social support (95% CI, −1.04 to −0.54, *p* < 0.001). This level of stress was also associated with stress about the lockdown, worries about lifestyle changes due to confinement (95% CI, 0.59 to 0.80, *p* < 0.001) and concerns about potential infection for a close relative (95% CI, −0.26 to −0.15, *p* < 0.001). A significant effect of media exposure on perceived stress was also found (95% CI, 0.174 to 0.520, *p* < 0.001). Regarding pre-lockdown addictive behaviors, a higher Cannabis Abuse Screening Test (CAST) (38) score (95% CI, 0.19 to 0.87, *p* = 0.002), Compulsive Internet Use Scale (CIUS) (39) score (95% CI, 0.08 to 0.14, *p* < 0.001) and SCOFF (33) score (95% CI, 0.36 to 0.57, *p* < 0.001) was also found for the mild stress group compared to the low stressed group.

With regard to the addictive behaviors displayed during lockdown, students reported more compulsive eating over the past week (95% CI, 0.48 to 0.79, *p* < 0.001) as well as more intention to do so in the next 15 days (95% CI, 0.65 to 1.49, *p* < 0.001) for the mild stress group compared to the low stress group. More restricted eating in the last week (95% CI, 0.2 to 0.44, *p* < 0.001) as well as more intention to restrict eating in the next 15 days (95% CI, 0.20 to 0.39, *p* < 0.001) and more intention to play online gaming in the next 15 days (95% CI, 0.01 to 0.01, *p* = 0.006) was also found for the mild stress group compared to the low stressed group.

#### The High Stress Group vs. the Low Stress Group

Similar results were found when the low stress students were compared to the most highly stressed students, with the exception that the high stress group generally held more scholarships (95% CI, 0.10 to 0.54, *p* = 0.004). In this population, the most stressed students had a greater number of relationships affected by COVID-19 (95% CI, 0.08 to 0.39, *p* = 0.003). In addition, their Alcohol Use Disorder test (AUDIT) scores (40) was higher (95% CI, 0.04 to 0.09, *p* < 0.001), which was strongly related to the level of perceived stress as well as the Internet Gaming Disorder Scale (IGDT10) (41) (95% CI, 0.06 to 0.13, *p* < 0.001) and Fagerström (30) scores (95% CI, 0.11 to 0.37, *p* < 0.001).

## Mixed-Effect Multinomial Logistic Regression Analysis to Identify Variables Linked With the Level of Perceived Stress

Using a multilevel mixed-effects multinomial model where all the previously significant variables were included as predictors, twelve independent variables were significantly associated with higher levels of stress (only comparisons between higher stress levels and lower stress levels are reported in this section; see Table 4 for more details). Students with a higher level of stress were more likely to be women (95% CI, 1.18 to 1.93, *p* < 0.001). The level of depression and anxiety was higher among the most stressed students (depression: 95% CI, 0.21 to 0.33, *p* < 0.001; anxiety: 95% CI, 0.43 to 0.54, *p* < 0.001), who also had less social support (95% CI, −1.04 to −0.39, *p* < 0.001). Highly stressed students were more likely to worry about the lockdown (95% CI, 0.27 to −0.65, *p* < 0.001). Additionally, alcohol-related problems were stronger among the most stressed students (AUDIT score: 95% CI, 0.02 to 0.09, *p* = 0.004) as well as eating problems (SCOFF score; 95% CI, 0.04 to 0.36, *p* = 0.016) and problematic use of the internet (CIUS score; 95% CI, 0.06 to 0.14, *p* < 0.001).

**Table 4:** Results of a multivariate analysis of factors related to students' perceived stress level.

	**Coeff**	**Std.Err**.	***z***	***p*-value**	**95% confidence interval**	
**Mild stress**						
_cons	−2.59	0.694	−3.73	<0.001	−3.95	−1.23
**Intrinsic characteristics**						
Gender	0.964	0.136	7.07	<0.001	0.697	1.231
Scholarship	0.019	0.117	0.16	0.872	−0.211	0.249
HADS-A	0.256	0.023	11.28	<0.001	0.212	0.301
HADS-D	0.106	0.026	4.14	<0.001	0.056	0.157
SPS10	−0.532	0.156	−3.42	0.001	−0.838	−0.227
**Addictive behaviors before lockdown**						
SCOFF	0.124	0.067	1.81	0.071	0.010	0.259
Fagerstrom	−0.060	0.076	−0.80	0.424	−0.209	0.088
AUDIT	0.032	0.015	2.13	0.033	0.003	0.061
IGTD10	0.016	0.025	0.62	0.538	−0.034	0.065
CIUS	0.064	0.016	3.87	<0.001	0.031	0.096
**Lockdown-specific scales**						
Stressors lockdown	0.290	0.071	4.08	<0.001	0.150	0.428
Stressors COVID19	0.042	0.047	0.88	0.380	−0.051	0.135
Having a loved one infected, hospitalized, or deceased because of COVID-19	0.076	0.086	0.89	0.375	−0.092	0.244
Media exposure	0.026	0.105	0.25	0.801	−0.180	0.233
**Addictive behaviors during lockdown**						
Compulsion next 15 days	0.515	0.232	2.22	0.026	0.060	0.969
Restriction last week	0.057	0.094	0.61	0.542	−0.127	0.242
Restriction next 15 days	−0.061	0.088	−0.70	0.487	−0.235	0.112
Online game next 15 days	0.005	0.002	2.13	0.033	0.001	0.010
**High Stress**						
_cons	−7.980	0.859	−9.29	<0.001	−9.664	−6.295
**Intrinsic characteristics**
Gender	1.557	0.192	8.10	<0.001	1.180	1.934
Scholarship	0.128	0.152	0.84	0.399	−0.169	0.425
HADS-A	0.486	0.027	17.94	<0.001	0.433	0.540
HADS-D	0.274	0.030	9.03	<0.001	0.214	0.333
SPS10	−0.671	0.191	−3.51	<0.001	−1.045	−0.296
**Addictive behaviors before lockdown**						
SCOFF	0.200	0.083	2.40	0.016	0.036	0.363
Fagerstrom	−0.139	0.095	−1.46	0.143	−0.324	0.047
AUDIT	0.052	0.018	2.88	0.004	0.017	0.088
IGTD10	−0.002	0.021	−0.06	0.954	−0.063	0.059
CIUS	0.101	0.021	4.84	<0.001	0.060	0.142
**Lockdown-specific scales**						
Stressors lockdown	0.459	0.095	4.82	<0.001	0.272	0.646
Stressors COVID19	0.023	0.063	0.37	0.709	−0.099	0.145
Having a loved one infected, hospitalized or deceased because of COVID-19	0.082	0.108	0.76	0.45	−0.131	0.295
Media exposure	−0.003	0.134	−0.02	0.984	−0.266	0.261
**Addictive behaviors during lockdown**						
Compulsion next 15 days	0.700	0.250	2.80	0.005	0.210	10.190
Restriction last week	0.006	0.112	0.05	0.957	−0.214	0.226
Restriction next 15 days	0.002	0.106	0.02	0.983	−0.206	0.211
Online game next 15 days	0.008	0.003	2.75	0.006	0.002	0.014

Regarding addictive behaviors during lockdown, higher stressed students had more compulsive eating during the last seven days (95% CI, 0.21 to 1.19, *p* = 0.005) and anticipated playing more online games in the next 15 days (95% CI, 0.00 to 0.01, *p* = 0.006) than the low stress group.

## Factorial Analysis

**Figure 1:**
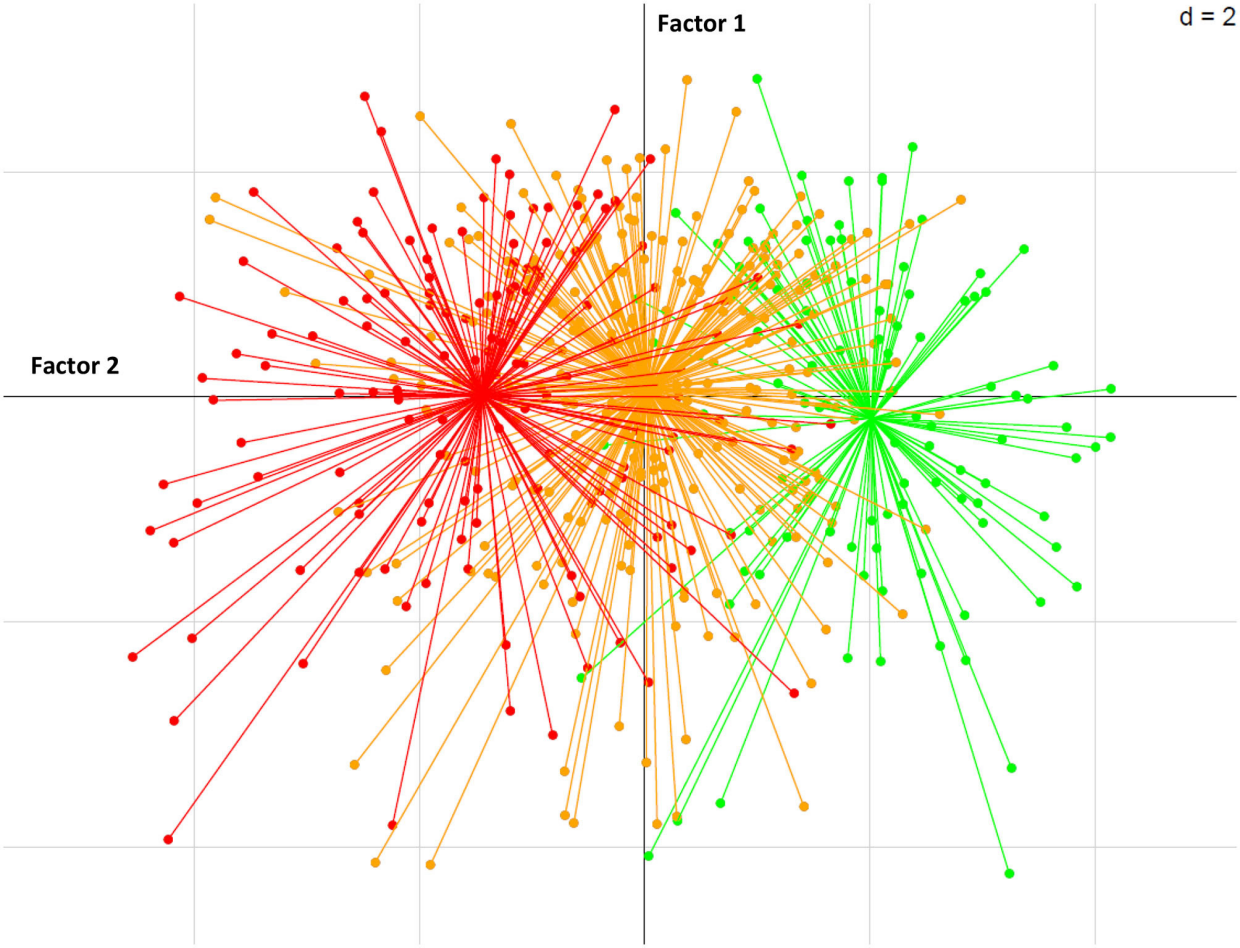
Three distinct profiles of students based on perceived stress level, illustrated by factorial analysis. The red color corresponds to the high-stressed student, the orange to the midly-stressed student and the green to the low-stressed student. The length of the arrows indicates the magnitude of the relationship to the PSS scores, so the longer the arrow, the greater the magnitude.

**Figure 2:**
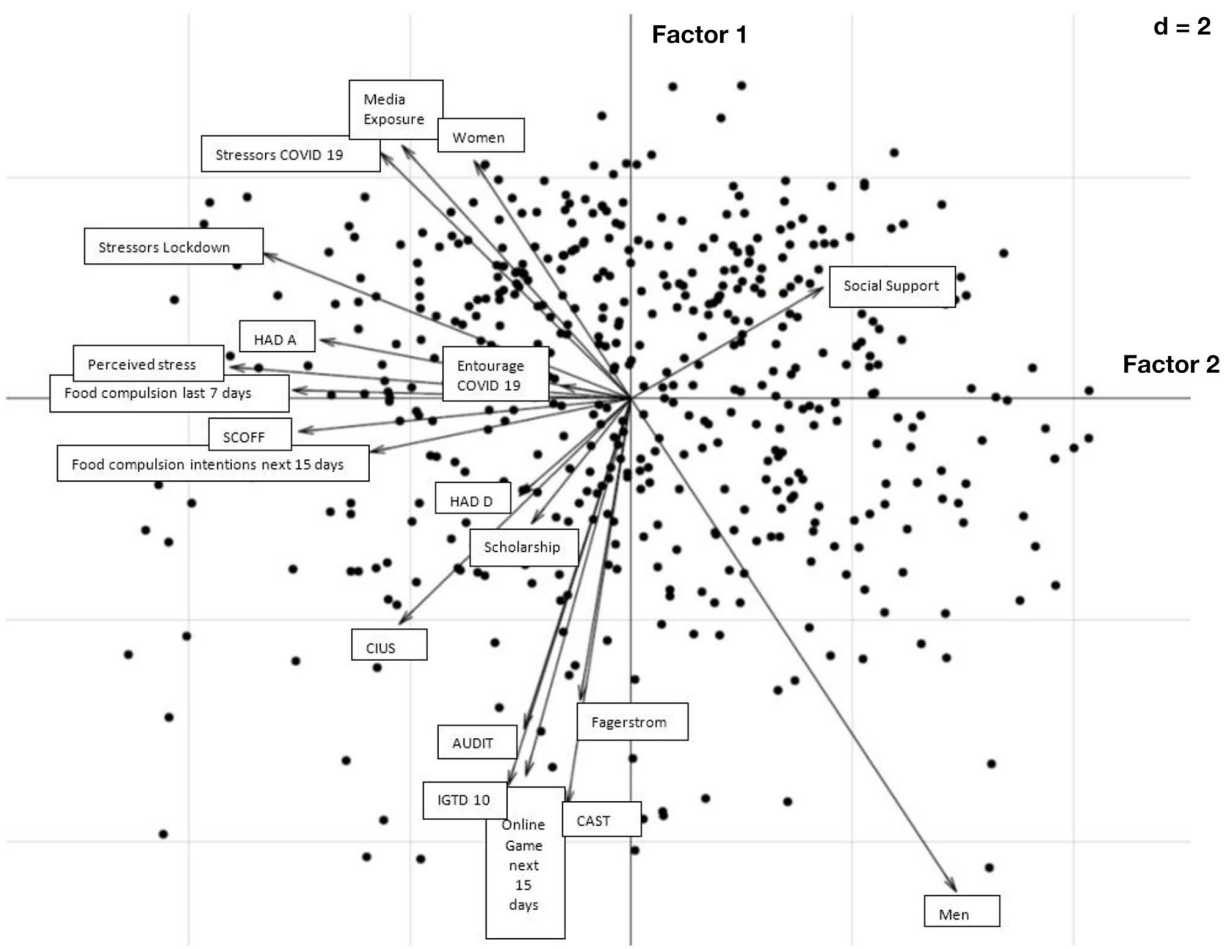
Variables of the perceived stress level induced by the lockdown and addictive behaviors before and during lockdown in the student population, illustrated by factorial analysis. The length of the arrows indicates the magnitude of the relationship to the different scales so the longer the arrow, the greater the magnitude.

Three distinct profiles of students based on perceived stress level were identified in a factorial analysis (see Figure 1). Based on this approach, concern about the lockdown, worry about a family member or friend becoming infected with COVID-19, media exposure and being female contributed to the highest perceived stress (see Figure 2). This proposed model represented 71% of initial information. This higher stress group was also associated with more anticipated compulsive eating next week, the intention to engage in compulsive eating over the next 15 days and the level of anxiety. Addictive behaviors before lockdown (measured by AUDIT, Fagerström, CAST and IGDT-10) and intention to game online over the next 15 days contributed to mild perceived stress, while a high level of social support contributed to the low perceived stress level.

## Discussion

The aim of this study was to assess the perceived stress related to the COVID-19 pandemic and lockdown in a sample of alcohol-drinking university students, assessing addictive behaviors linked with perceived stress before and during lockdown. Our results showed that students were particularly stressed during this period: more than 79% indicated having difficulty managing stress. The level of stress was strongly related to four categories of variables: (i) intrinsic characteristics, (ii) addictive behaviors before lockdown, (iii) lockdown-specific conditions, and (iv) addictive behaviors during the lockdown. A factorial analysis distinguished three different group of students by their level of perceived stress based on a number of variables.

The level of perceived stress in this population is higher than what was reported in other studies conducted in the same age group, further emphasizing the impact of the pandemic context on mental health. One previous study conducted between 2009 and 2011 on a population of 1,876 students in France found that 25% of students had a moderate or high level of stress (37). It is striking to note that 75% of our population demonstrated a moderate or high perceived stress level. Our results are consistent with other studies that have collected data over a similar period, but in other countries around the world and on non-student populations. Notably, Kowal et al. (42) observed that being a woman, living in a collectivist culture, being single and living with children were associated with higher levels of stress. Higher stress in women appears to be observed robustly in other work (40).

Women reported a higher level of stress than men, underscoring the fact that they are at increased risk for psychopathology and maladaptive coping behavior (e.g., substance abuse). Women reported frequently more sensitive to stress and negative affect than men (38) but are less likely to use psychoactive substances to cope with stress (43). In addition, women can be more sensitive to reduced social support when social norms change substantially. Previous research has suggested that reducing tension associated with stress is a motivating factor for alcohol use (44, 45), and that this relationship may differ by gender (38). Gender schema theory, which asserts that individuals are socialized to adopt behaviors they perceive as gender congruent (39, 43), suggests that while men are encouraged to engage in alcohol use women are expected to use it less. Under the stressful conditions of the pandemic, women may be able to respond to stress better via a pathological increase in food intake while men respond with increased alcohol consumption (46). This strategy may be augmented as social support is weakened (47). These results must be tempered by the fact that lower alcohol consumption among women under stressful and pandemic conditions is not a certainty. Recently Rodriguez et al. (44) suggested that psychological distress related to the COVID-19 pandemic was consistently related to alcohol use indices, significantly among women for number of heavy drinks. what should attract your attention in the Rodriguez study with respect to ours is that the average age (higher in this population [41.7 years of age (SD = 10.39)] as well as having children are risk factors for this use of alcohol.

Social support appears to be a major factor for resistance to stress. We observed that students with a higher level of social support experience lower stress levels. This is consistent with a recent study showing that the quality of offline social support constitutes a protective factor toward the development of excessive internet and social network involvement (45). Stress is therefore also dependent on the availability of social support and the effectiveness of coping strategies, (48). Hence, social support seems to be a plausible protective factor during lockdown.

During the first week of lockdown stress levels were not related to the level of financial precariousness of the students; whether or not a student had a scholarship for financial need had no effect on perceived stress. This might seem surprising since numerous studies have shown that social rank determines the rate of exposure to stressors (48). However, it is likely that this type of effect on stress could occur with a more prolonged stressful situation, and may be explained by the fact that this study was conducted in the first week of lockdown. A study exploring stress after several weeks of confinement could provide additional information on this.

Stress variables related to pursuing studies during the lockdown, such as worry about not being able to work or not succeeding professionally, were especially linked to the level of perceived stress. These conditions highlight the weight of the pandemic's uncertainties over the course of the academic year and the future of the student.

The level of perceived stress was not related to fears of contracting the disease. Similarly, perceived stress was not related to family or friends infected, hospitalized, or deceased from COVID-19. However, since the survey was conducted at the beginning of the confinement period, we cannot exclude that the number of people affected by COVID-19 was not large enough to sufficiently impact stress levels. The perceived stress of students is therefore more strongly related to the anticipation of consequences than to the actual consequences. Unexpectedly, media exposure to COVID-19-related information was not related to students' perceived stress levels.

In this study, we observed an effect of previous alcohol abuse on the level of perceived stress. These results are coherent with existing literature which has found that young adults with alcohol use disorders have more difficulty with stress management (49). However, there was no effect of tobacco consumption on the level of perceived stress. Additionally, cannabis use was not related to stress for students reporting using alcohol. Concerning addictive behaviors, heavy internet use was related to the perceived stress of students, which is in line with the compensatory internet use theory, which suggests that excessive involvement in online applications is displayed to escape negative emotions and psychopathological symptoms (50). Students consuming alcohol with dietary problems were also more sensitive to stress. Results showed that the level of perceived stress was strongly associated with a higher number of compulsive eating episodes in the previous week, suggesting that problematic eating can constitute a maladaptive coping strategy in a lockdown context. These results are in agreement with our study published on the same set of data, but on all students [see Flaudias et al. (22)]. Thus, one issue to consider is whether and under what conditions confinement associated with high stress can promote compulsive eating.

This study has several limitations. First, it is cross-sectional and does not allow for testing causal effects. Secondly, we explored our research questions with questionnaires created for the occasion and therefore without validation. We cannot exclude that the results could be different depending on the questions asked regarding the issues related to the pandemic. In addition, the participants are self-selected, which may have led to recruitment bias and therefore may not be representative. This choice was made based on self-regulation theories with a particular emphasis on the direct effect of alcohol on regulating capacities. Although this was not the focus of this paper, future research should not to limit oneself to this criterion limiting this selection bias. Nevertheless, the consumption data provided remains consistent with those found in national data (13). Finally, however, it is possible that including the covariates related to past problematic behaviors covered enough of the variance in common with our consumption measurements over the past week to statistically mitigate the effect.

To conclude, this study of student alcohol users shows that several variables linked to COVID-19 do not seem to be directly linked with perceived stress; however, stressors commonly linked to COVID-19 lockdown conditions (e.g., income and employment prospects, access to basic necessities, no access to social activities, etc.) were strongly associated with perceived stress. The increase in compulsive eating that students reported during lockdown suggests that students suffering from eating disorders constitute a high-risk population requiring more psychological support during and after the lockdown period. It is therefore urgent to implement preventive measures for this specific population to reduce the risk of persistent harmful eating habits once the pandemic has been resolved, especially for women, who are severely impacted by high stress.

## Data Availability Statement

The raw data supporting the conclusions of this article will be made available by the authors, without undue reservation.

## Ethics Statement

The studies involving human participants were reviewed and approved by ethics Committee of the University of Clermont Auvergne. The patients/participants provided their written informed consent to participate in this study.

## Author Contributions

VF, OZ, MN, and GB participated to study concept, design, analysis, interpretation of data, statistical analysis, study supervision, wrote the initial draft of the article, and full access to all data in the study and take responsibility for the integrity of the data and the accuracy of the data analysis. BP and IC done analysis. P-ML, IC, LR, LS, LB, MN, and GB participated to analysis and interpretation of data. P-ML, IC, LR, LS, LB, CC, JB, SG, JC, LG, and BR reviewed the initial draft and participated in the writing of the final draft. All authors contributed to the article and approved the submitted version.

## Conflict of Interest

The authors declare that the research was conducted in the absence of any commercial or financial relationships that could be construed as a potential conflict of interest.

## Footnotes

^1^Here, technical guidance refers to advice given to the population in France to curb the pandemic at the level of individual behaviors (e.g., coughing into one's elbow, respecting social distance, how to put on a mask), while public policies refers to collective aspects of health policy (e.g., lockdown, telecommuting, closing of restaurants, and non-essential shops).
