# Supplementary material for: The Early Impact of the COVID-19 Lockdown on Stress and Addictive Behaviors in an Alcohol-Consuming Student Population in France
Source: Front Psychiatry. 2021 Feb 9;12:628631. doi: 10.3389/fpsyt.2021.628631 (PMC7900161; doi:10.3389/fpsyt.2021.628631)
Supplement: Supplementary file 1 [file Data_Sheet_1.docx]

APPENDIX A – Self-reported questionnaires

**Environmental stressors**

*Instructions: We're going to ask you a few things that can be stressful during Lockdown. Again, there is no right or wrong answer, we are only interested in your sincere answer. Participants HADS to answer on a scale from 1 (Strongly Agrees) to 7 (Strongly Disagrees)*

1. My daily income
2. Being able to do my job well enough
3. Future employment prospects
4. Access to basic necessities such as food
5. Not being able to participate in group social activities
6. Dealing with the behavior of adults with whom I isolate myself
7. Dealing with the behavior of children with whom I isolate myself
8. The national economy
9. The risk that I or others I know will catch COVID-19
10. The risk of myself or others I know being hospitalized or dying because of COVID-19
11. Adapting your work to digital platforms
12. Having to adapt one's social life to digital platforms
13. Being ashamed to act differently from others (e.g. at work, shopping)

**Condition of lockdown** Instructions: *Indicate what best describes your current situation*

- If you are locked down, how many other adults live together in the same place as you?
- How many people live in the city where you live?
- What is the size of the place where you live during Lockdown (in m²)?
- Does your living location have an exterior?

o Yes

o No

- If yes, do you have access to a:

o Balcony

o Garden

o Inner courtyard

- If you are locked down, how many children under the age of 12 live together in the same place as you?
- Do you know of anyone infected with Covid-19?

o Yes

o No

- Do you know anyone who's been hospitalized because of Covid-19?

o Yes

o No

- Do you know people who died because of Covid-19?

o Yes

o No

**APPENDIX B – Self-report consumption questionnaires**

**Alcohol questions**

- In the past week, what was your alcohol consumption?

o Once a week

o 2 to 3 times a week

o Every day or almost every day

o None

- If so, how many standard drinks do you have on a typical drinking day?
- Do you plan to consume alcohol in the next 15 days?

o Yes

o No

- If yes

o once a week

o 2 to 3 times a week

o Every day or almost every day

- How many standard glasses do you think you'll consume on a typical drinking day (see picture)?

**Binge drinking questions**

- Do you plan to drink six or more standard glasses on one occasion in the next 15 days?

o Yes

o No

- If so, how many times?
- Over the past week, have you ever consumed more than 7 drinks of alcohol in less than 2 hours if you are a man or more than 6 drinks if you are a woman?

o Yes

o No

- Was this consumption in a "virtual meeting"?

o Yes

o No

- How many times has this type of event (consumption of more than 7 or 6 drinks in less than 2 hours) occurred in 7 days?

**Food Compulsion**

- In the past week have you had bouts of compulsive eating (heavy food intake over a short period of time)?

o None

o Less than once a week

o 2 to 3 times a week

o Almost every day

- Do you plan to stockpile food in the next 15 days for possible compulsive eating?

o No

o less than once a week

o 2 to 3 times a week

o almost every day

**Food Restriction**

- In the past week have you had any voluntary food restrictions due to weight or figure concerns?

o none

o less than once a week

o 2 to 3 times a week

o almost every day

- Do you plan in the next 15 days to voluntarily restrict your eating for weight or figure concerns?

o No

o less than once a week

o 2 to 3 times a week

o almost every day

**Online Gaming**

*Two more questions about your use of online games:*

Overall, would you say that in the last 7 days your use of online games has increased? (From 0 – Not at all to 100 Totally)

Do you think your use of online games will increase in the next 15 days? (From 0 – Not at all to 100 Totally)

**Cannabis**

- In the past week, what was your cannabis use?

o None

o Less than once a week

o 2 to 3 times a week

o Several times a week

- If yes, how many joints of cannabis did you consume on a typical day when you smoked?
- Do you plan to use cannabis in the next 15 days?

o No

o Less than once a week

o 2 to 3 times a week

o Several times a week

- If yes, how many joints of cannabis do you think you will consume on a typical day when you go out for a smoke?
